# Diagnostic performance of FIB-4 in detecting advanced liver fibrosis in patients with rheumatoid arthritis, psoriatic arthritis and psoriasis using methotrexate

**DOI:** 10.1186/s42358-026-00567-7

**Published:** 2026-07-24

**Authors:** Fernanda Luiza Schumacher Furlan, Patricia Martin, Alcindo Pissaia Junior, Claudia Alexandra Pontes Ivantes, Marcia Olandoski, Adriane Reichert Faria, Adriano Reinaldo Timm, Jhony Maciel de Oliveira, Giuliano Camati Dourado, Lana Bassi Ferdinando, Rebeca Rebouças Pereira, Beatriz Gallo Teixeira, Raphaela Suzin Marini Munaretto, Odery Ramos Junior

**Affiliations:** 1 Department of Internal Medicine, Universidade Federal do Paraná, Curitiba, Brazil; 2 Department of Gastroenterology, Hepatology and Liver Transplantation, Hospital Nossa Senhora das Graças, Curitiba, Brazil; 3 School of Medicine and Life Sciences, Pontifícia Universidade Católica do Paraná, Curitiba, Brazil; 4 Faculdade Evangélica Mackenzie de Medicina do Paraná, Curitiba, Brazil; 5 Department of Internal Medicine, Pontifícia Universidade Católica do Paraná, Curitiba, Brazil; 6 Department of Dermatology, Hospital Santa Casa de Misericórdia, Curitiba, Brazil; 7 Department of Internal Medicine, Hospital Municipal de São José dos Pinhais, Curitiba, Brazil

**Keywords:** Liver fibrosis, Ultrasonography, Elastography, Rheumatoid arthritis, Psoriatic arthritis, Psoriasis, Methotrexate

## Abstract

**Background:**

Methotrexate is a first-line therapy for rheumatoid arthritis, psoriatic arthritis, and psoriasis, but its long-term use has been associated with hepatic fibrosis, highlighting the need for effective screening strategies. In this context, and considering the limitations of liver biopsy as well as the high cost and limited availability of elastography, the FIB-4 score has emerged as the most widely used noninvasive screening tool. This multicenter cross-sectional study evaluated patients followed at specialized Rheumatology and Dermatology centers to assess the accuracy of FIB-4 in predicting advanced hepatic fibrosis in patients with rheumatoid arthritis, psoriatic arthritis, and psoriasis undergoing chronic methotrexate therapy. The specific objectives were to identify optimal cutoff values for FIB-4 in this population and factors associated with advanced fibrosis.

**Methods:**

Medical records, interviews, and physical examinations were used to collect demographic, clinical, laboratory, lifestyle, and treatment data, calculate age-adjusted FIB-4 scores, and assess disease activity using validated indices. Liver fibrosis and steatosis were evaluated by vibration-controlled transient elastography, and FIB-4 performance for detecting advanced fibrosis was analysed using age-stratified Receiver Operating Characteristic curves and multivariable logistic regression.

**Results:**

A total of 172 patients were included, predominantly women, with a mean age of 58 years; rheumatoid arthritis was the most common underlying disease. Advanced fibrosis was identified by elastography in 11%. FIB-4 values increased progressively with fibrosis stage, and Receiver Operating Characteristic curve analysis identified a cutoff of 1.62 — a threshold not previously reported for methotrexate-treated patients. This cutoff demonstrated better performance in individuals younger than 65 years (Area Under the Curve = 0.68 *versus* 0.64 in those aged 65 years or older) and showed particular utility for ruling out advanced fibrosis. In multivariable analysis, a high-risk FIB-4 category, obesity, *diabetes mellitus*, and advanced hepatic steatosis were independently associated with advanced fibrosis, whereas inflammatory disease activity and cumulative methotrexate dose were not.

**Conclusions:**

FIB-4 is an accessible and potentially useful tool for ruling out advanced hepatic fibrosis in methotrexate-treated patients younger than 65 years, demonstrating reasonable performance in this population. Advanced fibrosis is primarily driven by metabolic factors rather than methotrexate exposure or inflammatory disease activity.

## Background

Rheumatoid arthritis (RA), psoriatic arthritis (PsA), and psoriasis (Pso) are prevalent diseases for which methotrexate remains a first-line therapy due to its proven efficacy and broad accessibility [1–3]; however, its long-term use has been associated with hepatotoxicity and liver fibrosis [4–6], prompting Rheumatology and Dermatology guidelines to recommend regular laboratory monitoring to mitigate liver-related morbidity and mortality [7–10]. Nevertheless, this risk may be overestimated due to confounding factors [5, 6]. 

Although liver biopsy remains the gold standard for diagnosing hepatic fibrosis, its invasiveness, associated risks, and susceptibility to observer and sampling bias limit its use [11–13]. Consequently, noninvasive assessment strategies, including serum-based scores and imaging methods, play an important role in clinical practice. Among these, vibration-controlled transient elastography (VCTE; FibroScan^®^) represents an effective alternative; however, it also has important limitations, such as operator dependence, erratic performance in obese individuals, high cost and limited availability [6, 14, 15].

In contrast, the Fibrosis-4 (FIB-4) score is an inexpensive and easily applicable noninvasive tool for assessing liver fibrosis based on routinely available serum markers and clinical parameters [1, 6, 11, 12, 16]. It has been validated across several chronic liver diseases, particularly metabolic dysfunction-associated steatotic liver disease (MASLD), where cutoffs of < 1.3 for individuals younger than 65 years and < 2.0 for those aged 65 years or older are commonly used to exclude advanced fibrosis [16]. Not only is the diagnostic performance affected by age, but also by the clinical context, particularly in systemic inflammatory rheumatic diseases, where inflammation and disease-modifying therapies can affect aminotransferase levels and platelet counts (variables involved in the calculation of the score) [6, 16]. Although FIB-4 is well established for liver fibrosis screening in metabolic diseases, it has not been specifically validated in patients with rheumatic diseases, in whom higher scores appear to reflect metabolic and cardiovascular risk rather than disease-specific features [5]. 

Recognizing the limited availability of elastography, the main objective of this study was to evaluate the accuracy and reliability of FIB-4 in predicting the risk of advanced liver fibrosis in patients with RA, PsA, and Pso undergoing chronic methotrexate therapy. The specific objectives were to evaluate FIB-4 cutoff values indicating risk of advanced fibrosis according to age groups (< 65 years and ≥ 65 years) and to identify the factors associated with advanced liver fibrosis.

## Methods

This observational, multicenter, cross-sectional study included both retrospective and prospective data from patients diagnosed with RA, PsA, or Pso from three specialized Rheumatology or Dermatology centers in Curitiba, Brazil.

Inclusion criteria were: age > 18 years, use of methotrexate for at least 6 months and a diagnosis of RA, PsA or Pso according to the classification criteria established by international Rheumatology and Dermatology societies [7, 17]. 

Exclusion criteria were: missing medical record data, active viral hepatitis under antiviral treatment, and significant alcohol consumption (> 1 standard drink/day for women and > 2 standard drinks/day for men) [18], as these conditions are well-established independent causes of liver fibrosis that could confound the assessment of methotrexate-associated liver injury.

Participants were invited to read and sign the Informed Consent Form, in accordance with the Research Ethics Committee (REC) of each institution where the project was approved.

Patients’ medical records were reviewed to collect data on current age, age at the time of diagnosis, duration of RA, PsA or Pso, concomitant medications in use, such as biologic therapy, small molecule drugs, oral corticosteroids or leflunomide, comorbidities, ethnicity, sex, rheumatoid factor positivity, laboratory test results (obtained within up to six months prior to elastography) and cumulative methotrexate dose. MASLD was defined according to multisociety criteria [19]. 

Subsequently, patients were interviewed about lifestyle habits, including current or previous smoking, alcohol consumption and physical activity. This was followed by a physical examination to record weight, height, waist circumference, blood pressure and clinical assessment of disease activity using validated indices according to the underlying condition (Disease Activity Score in 28 joints - DAS28 [17]; Disease Activity Index for Psoriatic Arthritis - DAPSA [9]; Psoriasis Area and Severity Index - PASI [7]).

The FIB-4 score was determined using validated online calculators based on the formula [20]: (age in years × AST value in U/L) / [platelet count (10⁹/L) × √ALT value in U/L]; AST = Aspartate Aminotransferase and ALT = Alanine Aminotransferase. Afterwards, FIB-4 risk stratification for advanced fibrosis was established according to the age, since cutoffs are age-specific [21]: in individuals < 65 years, values < 1.30 indicate low risk; 1.30–2.67, an indeterminate risk; and > 2.67, high risk of advanced fibrosis; in those ≥ 65 years, values < 2.0 are considered low risk; 2.0–2.67, indeterminate; and > 2.67 high risk.

After clinical evaluation, patients were referred to a specialized Hepatology center in Curitiba for VCTE using FibroScan 502 Touch^®^ or FibroScan Mini + 430^®^ devices (Echosens, Paris, France). The result of liver stiffness was expressed in kilopascals (kPa) and established by the median of at least 10 valid measurements. Results were considered reliable when the ratio of the interquartile range (IQR) to the median was less than 30% [22]. Examinations exceeding this threshold were repeated to ensure adequacy. Five fibrosis stages were defined, based on the classification proposed by Tsochatzis et al., 2011 [23]: F0 (< 6.5 kPa), F1 (6.5–7.1 kPa), F2 (7.2–9.5 kPa), F3 (9.6–14.4 kPa), and F4 (≥ 14.5 kPa), with advanced liver fibrosis defined as stage F3 or F4. Hepatic steatosis was classified into four grades, based on the values proposed by Karlas et al., 2017 [24].: S0 (< 248 CAP), S1 (248–267 CAP), S2 (268–279 CAP), and S3 (≥ 280 CAP). Data obtained from the examinations were stored in a Microsoft Excel^®^ spreadsheet.

Quantitative variables were described as mean, standard deviation, median and interquartile interval, whereas categorical variables were presented as absolute and relative frequencies.

To determine FIB-4 cutoff values associated with the presence of advanced fibrosis, defined as F3/F4 on VCTE, Receiver Operating Characteristic (ROC) curves were constructed and the areas under the curve (AUCs) were calculated. ROC analyses were stratified according to age (< 65 years and ≥ 65 years), because FIB-4 thresholds for fibrosis risk differ between these age groups, as previously described. Optimal cutoff points were defined using the Youden index. For each cutoff, sensitivity, specificity, positive predictive value (PPV), and negative predictive value (NPV) were estimated, considering the prevalence of advanced fibrosis observed in the study sample.

Associations between clinical and laboratory variables and the probability of advanced fibrosis were evaluated using univariable and multivariable logistic regression models. FIB-4 was analyzed according to risk category, comparing the high-risk category with the low-risk / gray-zone categories. Given the limited number of advanced fibrosis cases (*n* = 19), a parsimonious modelling strategy was adopted to reduce the risk of overfitting and model instability. Therefore, instead of fitting a single multivariable model including all potential confounders simultaneously, separate logistic regression models were constructed, each including FIB-4 and one selected demographic, clinical, or laboratory covariate. Covariates were selected based on univariable analysis (*p* < 0.25) and clinical relevance. This approach was intended to assess whether the association between high FIB-4 and advanced fibrosis persisted after individual adjustment for potential confounding factors, rather than to determine the fully independent contribution of each covariate.

The statistical significance of associations was assessed using the Wald test, and measures of association were expressed as odds ratios (ORs) with their respective 95% confidence intervals (95% CIs). A two-sided significance level of 5% (*p* < 0.05) was adopted. To account for the potential inflation of type I error due to multiple individually adjusted models, *p*-values for the association between high FIB-4 and advanced fibrosis across these models were additionally adjusted using the Benjamini-Hochberg false discovery rate (FDR) procedure.

This study included all eligible patients recruited from 2021 to 2025. No formal a priori sample size calculation was performed. Although the final sample allowed the detection of a statistically significant association between high FIB-4 and advanced fibrosis in the main analysis, subgroup analyses according to age and disease type were considered exploratory because of the limited number of advanced fibrosis cases.

Statistical analyses were performed using Microsoft Excel^®^ for data organization and IBM SPSS Statistics, version 30.0.0 (IBM Corp., Armonk, NY, USA), for statistical analyses.

This study was approved by the REC of each institution, in accordance with the requirements of Resolution 466/12 of the Brazilian National Health Council. Approval numbers: 7.922.034, 7.933.624 and 7.883.040.

## Results

### Characterization of the sample

A total of 176 individuals meeting the eligibility criteria for inclusion in the study were initially identified. Four were subsequently excluded: 2 of them due to changes in the diagnosis (osteoarthritis and Primary Sjögren’s Disease), 1 due to death prior to undergoing elastography, and 1 due to morbid obesity, which prevented a proper FibroScan^®^ performance. The final sample consisted of 172 participants, most of which were women (82%), caucasian (82%), with a mean age of 58.1 ± 12.3 years. Individuals with less than 65 years of age represented 68.6% of the sample (*n* = 118).

Regarding the diagnosis, most had RA (79.1%), followed by PsA (15.7%) and Pso (5.2%). The mean age at diagnosis was 47.1 ± 14.6 years, and the mean disease duration was 10.9 ± 8.5 years. Rheumatoid factor was positive in 53.5% of patients, and most individuals (71.0%) were in remission or had low disease activity.

In respect of the treatment, the mean cumulative methotrexate dose was 6.5 ± 5.2 g. Concomitant use of prednisone was reported in 26.2% of participants and of leflunomide, in 32.6%. Also, biologic agents or small molecule drugs were used in 26.2%. Of these, more than half (55.1%) were anti-tumor necrosis factor (anti-TNF) therapy (adalimumab, certolizumab, etanercept, golimumab and infliximab). The other agents in use were baricitinib, tofacitinib, upadacitinib, abatacept, secukinumab and tocilizumab.

The most prevalent comorbidity was MASLD (59.3%), followed by systemic arterial hypertension (48.8%), dyslipidemia (43.6%), obesity (41.5%), depression / anxiety (34.9%), type 2 *diabetes mellitus* - T2DM (22.7%), hypothyroidism (21.5%), and osteoarthritis (13.4%).

The individuals were predominantly overweight, with a mean body mass index (BMI) of 29.4 ± 6.4 kg/m². It was also observed that most had an increased waist circumference measurement, with a mean measurement of 105.12 ± 19.64 cm in the men and 95.52 ± 14.2 cm in the women evaluated.

As to the lifestyle habits, 33.7% were current or former smokers, with a mean smoking exposure of 24.8 ± 30.2 pack-years. Additionally, regular physical activity was reported by only 23.8% of participants. Alcohol consumption was reported in 5.2%, and none consumed amounts considered potentially hepatotoxic in conformity with the exclusion criteria.

Regarding the laboratory tests, it was observed that only the mean value of glycated hemoglobin (HbA1c) was altered in comparison to its normal range: 5.7 ± 0.85%.

The data of FIB-4 and elastography were summarized in Table 1. Notably, advanced fibrosis (F3–F4 by elastography) was identified in 19 individuals, corresponding to 11% of the study population.

**Table 1:** FIB-4 and elastography data

Variable	Classification	*n*	Result
FIB-4	Mean ± SD Median (min–max)	172	1.68 ± 1.21 1.40 (0.21–8.36)
FIB-4 classification according to advanced fibrosis risk¹	Low	102	59.3%
	Gray zone	48	27.9%
	High	22	12.8%
FIB-4 dichotomous risk classification²	Low	102	59.3%
	Gray zone / High	70	40.7%
Liver stiffness in kPa (Elastography)	Mean ± SD Median (min–max)	172	6.7 ± 5.7 5.2 (2.5–56.4)
Fibrosis stage (Elastography)³	0	120	69.8%
	1	12	7.0%
	2	21	12.2%
	3	11	6.4%
	4	8	4.7%
Advanced fibrosis (Elastography F3/F4)	No	153	89.0%
	Yes	19	11.0%
Hepatic steatosis in CAP (Elastography)	Mean ± SD Median (min–max)	172	261 ± 61.1 260 (100–400)
Steatosis grade (Elastography)⁴	0	69	40.1%
	1	27	15.7%
	2	15	8.7%
	3	61	35.5%

Abbreviations: FIB-4: Fibrosis-4; SD: Standard Deviation; min: minimum; max: maximum; kPa: kilopascals; F3: liver fibrosis stage 3; F4: liverfibrosis stage 4; CAP: Controlled Attenuation Parameter

Notes: 1,2: The FIB-4 risk classification was determined by the following criteria: <65 years of age: <1.30: low risk of significant fibrosis / 1.30–2.67:gray zone / indeterminate / >2.67: high risk of advanced fibrosis

3: The grades of fibrosis on elastography were classified as: F0 (< 6.5 kPa), F1 (6.5–7.1 kPa), F2 (7.2–9.5 kPa), F3 (9.6–14.4 kPa), and F4 (≥ 14.5 kPa)

4: The grades of steatosis on elastography were classified as: S0 (< 248 CAP), S1 (248–267 CAP), S2 (268–279 CAP), and S3 (≥ 280 CAP)

### Assessment of the association between FIB-4 and the presence of advanced fibrosis on elastography (F3/F4), considering FIB-4 as a quantitative variable

**Fig. 1:**
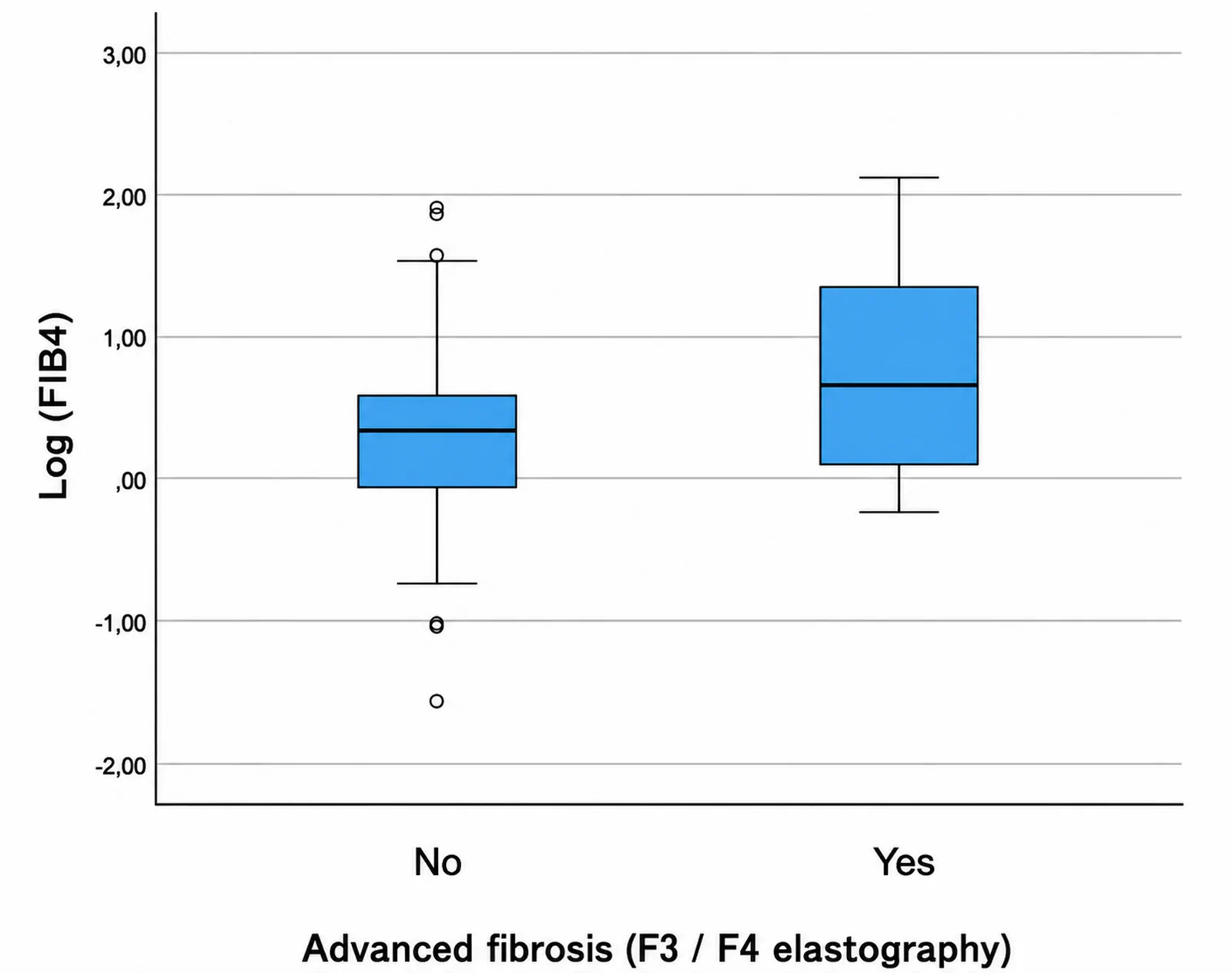
Comparison between FIB-4 (log) and advanced fibrosis (elastography). Boxplot comparing log-transformed FIB-4 values according to the presence or absence of advanced liver fibrsosis (F3/F4) as assessed by elastography. Boxes represent the interquartile range with the median indicated by the horizontal line; whiskers denote dispersion, and dots indicate outliers. Higher log (FIB-4) values are observed in individuals with advanced fibrosis. Source: The Authors (2026)

Initially, the sample was stratified according to the presence of advanced liver fibrosis, defined by elastography as stages F3 or F4. Differences in mean FIB-4 values between patients with and without advanced fibrosis were assessed by testing the null hypothesis of equal means versus the alternative hypothesis of unequal means. Comparisons were performed using Student’s *t*-test for independent samples. Patients without advanced fibrosis presented a mean FIB-4 of 1.54 ± 0.96 and a median of 1.40 (range: 0.21–6.73), whereas those with advanced fibrosis showed a higher mean FIB-4 of 2.74 ± 2.18 and a median of 1.93 (range: 0.79–8.36). The difference was statistically significant (*p* < 0.001), leading to rejection of the null hypothesis and indicating that FIB-4, as a continuous variable, effectively discriminates between patients with and without advanced liver fibrosis. The comparison between log-transformed FIB-4 values and elastography-defined advanced fibrosis (F3/F4) is shown in Fig. 1.

### Determination of FIB-4 cutoff values considering elastography results (ROC curves) according to the age group (< 65 or ≥ 65 years)

**Fig. 2:**
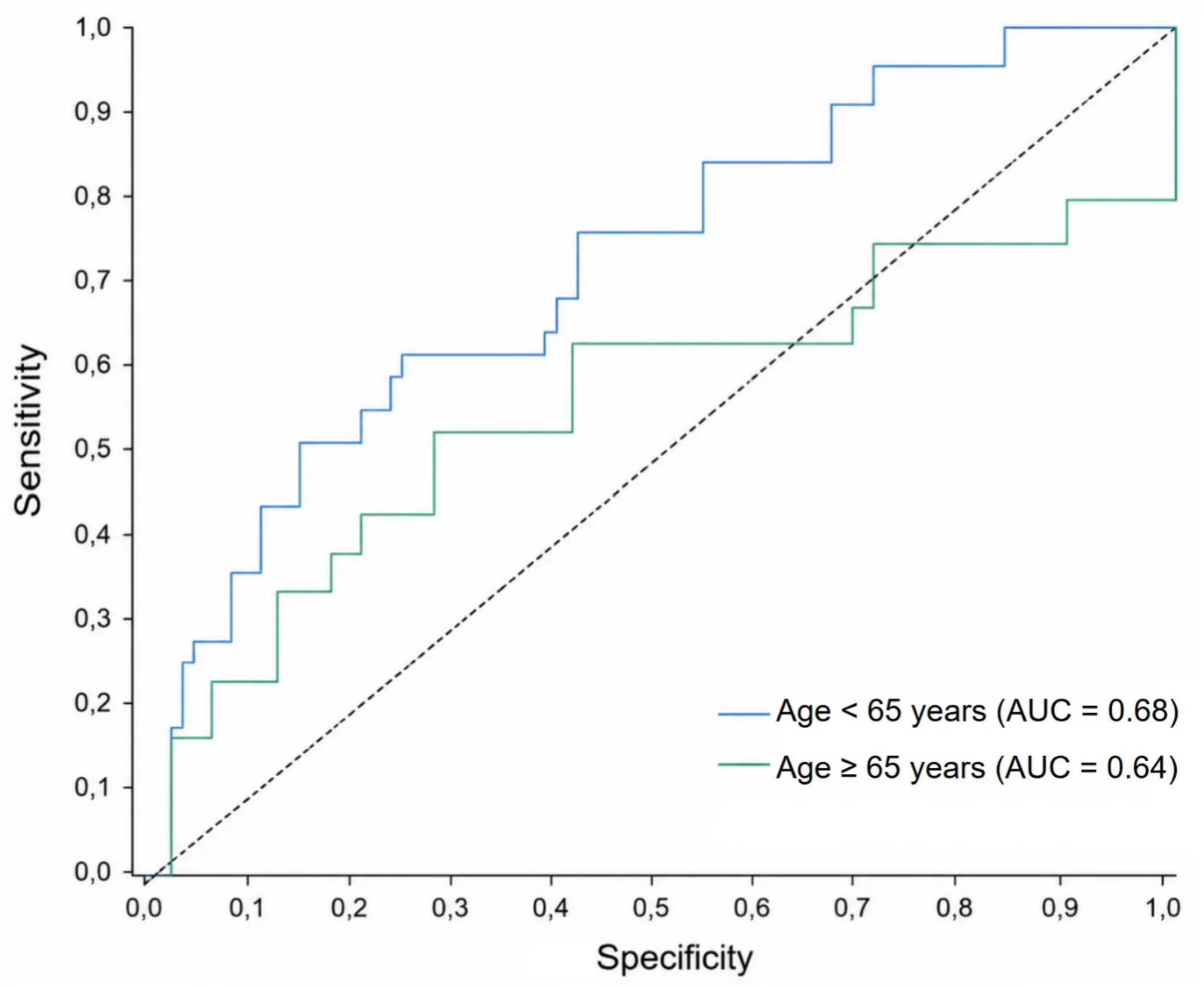
Performance of FIB-4 in predicting advanced fibrosis (elastography) in each age group. Receiver operating scharacteristic (ROC) curve illustrating the performance of FIB-4 in predicting advanced liver fibrosis (Fs3/F4) assessed by elastography among individuals aged < 65 years and ≥ 65 years. Sensitivity is plotted against specificity, with the diagonal dashed line representing the linse of no discrimination. The curves demonstrate the discriminative ability of FIB-4 for identifying advanced fibrosis in each age group. Source: The Authors (2026)

To determine FIB-4 cutoff values capable of discriminating between the presence or absence of advanced fibrosis on elastography (F3/F4), ROC curves were elaborated and the areas under the curve (AUC) were evaluated. The cutoff values were then defined using the Youden index. This analysis was performed stratified by age (< 65 or ≥ 65 years) and displayed in Fig. 2.

The analysis for individuals < 65 years indicated that FIB-4 discriminated well between the presence or absence of advanced fibrosis on elastography in this age group. The AUC was 0.68, with statistical significance (*p* = 0.045). The cutoff value identified by the model was FIB-4 = 1.62 (values below 1.62 indicate that advanced fibrosis is unlikely).

For individuals aged ≥ 65 years, FIB-4 did not adequately discriminate between the presence and absence of advanced fibrosis on elastography. The AUC was 0.64 and did not reach statistical significance (*p* = 0.327); therefore, no cutoff value was established in this age group.

### Diagnostic performance of the 1.62 cutoff value in discriminating advanced fibrosis according to elastography (F3/F4)

Performance metrics for the 1.62 cutoff value, including sensitivity, specificity, accuracy and predictive values, were also calculated separately for each age group (< 65 years and ≥ 65 years). Among individuals < 65 years (Table 2), the 1.62 cutoff demonstrated good overall performance, with an accuracy of 76.3% and a high negative predictive value (94.4%).

**Table 2:** Evaluation of the quality of the cutoff point of 1.62 in the age group of < 65 years

Quality of the 1.62 cut-off point for age < 65 years	Result (%)	95% CI (%)
Sensitivity	54.5	25.1–84.0
Specificity	78.5	70.7–86.3
Accuracy	76.3	68.6–83.9
PTP+	21.5	13.7–29.3
PTP-	45.5	16.0–74.9
PPV	20.7	5.9–35.4
NPV	94.4	89.6–99.2

Abbreviations: 95% CI: Confidence Interval of 95%; PTP+: Post-test positive probability; PTP−: Post-test negative probability; PPV: PositivePredictive Value; NPV: Negative Predictive Value

Note: For the calculation of PPV and NPV, the prevalence of advanced fibrosis was estimated based on the study sample (9.3%)

In patients aged ≥ 65 years, despite demonstrating a higher sensitivity than the other age group (75.0%, with a 95% CI of 45–100%), the same cutoff value proved to be much less specific (34.8%, 95% CI 21-48.5%) and less accurate (40.7%, 95% CI 27.6–53.8%), generating a high number of false positives. Therefore, the overall performance was poor in this age group.

### Variables associated with advanced hepatic fibrosis (elastography F3/F4)

Potential variables associated with advanced hepatic fibrosis were initially assessed using univariate logistic regression analysis. For each variable, the null hypothesis of no association with the probability of advanced fibrosis was tested against the alternative hypothesis of an existing association. OR with corresponding 95% CI and *p* values were estimated. For categorical variables with more than two levels, one category was designated as the reference group, and the remaining categories were compared to it. Statistical significance of associations was evaluated using the Wald test.

The potential variables associated with advanced hepatic fibrosis identified were: BMI, obesity, T2DM, systemic arterial hypertension, osteoarthritis, waist circumference, fasting glucose, HbA1c, AST, ALT, platelet count, FIB-4 as a quantitative variable and FIB-4 classified into risk categories, as well as age at diagnosis (the older, the bigger risk). Among these, the strongest potential variable was T2DM (OR 10.6; 95% CI 3.68–30.4).

Use of biologic agents, small molecule drugs, corticosteroids or leflunomide were not potential variables associated with advanced fibrosis, nor were the cumulative methotrexate dose, disease activity, or the underlying disease (RA / PsA / Pso), as shown by *p* values > 0.05.

Considering that only 19 cases of advanced fibrosis identified by elastography (F3/F4) were observed, multivariable logistic regression models were constructed to evaluate the association between FIB-4 and this outcome while minimizing the risk of overfitting and model instability. In each model, FIB-4 was paired with one demographic or clinical variable selected from the univariate analysis (*p* value < 0.25) and considered clinically relevant. This parsimonious modelling strategy was adopted because the limited number of outcome events did not allow the simultaneous inclusion of multiple correlated covariates, particularly metabolic variables such as obesity, waist circumference, steatosis grade, MASLD, and T2DM. Therefore, these models were intended to assess whether the association between FIB-4 and advanced fibrosis persisted after individual adjustment for potential confounding factors, rather than to determine the independent contribution of each metabolic variable. The results are presented in Table 3.

**Table 3:** Association between high FIB-4 (high risk x low risk / gray zone) and advanced liver fibrosis after individual adjustment for selected covariates

Covariate included in the model with FIB-4	*p*-value for FIB-4*	Adjusted OR for high FIB-4 (95% CI)	Benjamini-Hochberg adjusted *p*-value
Sex (female x male)	0.002	5.58 (1.87–16.7)	0.003
Disease duration (years)	0.002	6.00 (1.98–18.2)	0.003
Mixed-race x Caucasian	0.001	6.03 (1.99–18.2)	0.002
BMI	< 0.001	13.4 (3.58–50.5)	0.002
Obesity	< 0.001	19.2 (3.77–98.2)	0.002
Steatosis grade (elastography)	< 0.001	13.4 (3.58–50.5)	0.002
Cumulative MTX dose (g)	0.004	5.49 (1.71–17.6)	0.005
T2DM	0.006	5.71 (1.66–19.7)	0.006
Systemic Hypertension	0.004	5.07 (1.66–15.5)	0.005
WC (cm)	< 0.001	19.1 (4.22–86.3)	0.002
HDL (mg/dL)	0.003	5.53 (1.81–16.9)	0.004
ESR (mm)	< 0.001	7.40 (2.25–24.3)	0.002
Moderate / high disease activity**	0.066	3.31 (0.92–11.8)	0.066
MASLD	< 0.001	8.15 (2.46–27.0)	0.002

Abbreviations: BMI: Body Mass Index; MTX: methotrexate; T2DM: Type 2 diabetes mellitus; WC: Waist circumference; HDL: High-densitylipoprotein; ESR: Erythrocyte sedimentation rate; MASLD: Metabolic dysfunction–associated steatotic liver disease

Notes: The FIB-4 risk classification was determined by the following criteria: <65 years of age: <1.30: low risk of significant fibrosis / 1.30–2.67:gray zone / indeterminate / >2.67: high risk of advanced fibrosis. ≥65 years of age: <2.0: low risk of significant fibrosis / 2.0–2.67: gray zone /indeterminate / >2.67: high risk of advanced fibrosis

*Multivariable logistic regression model and Wald test, p < 0.05. Benjamini-Hochberg correction was applied to the p-values for high FIB-4 across theindividually adjusted models. For p-values reported as < 0.001, a conservative value of 0.001 was used for the correction

**Disease activity was assessed using validated indices according to the underlying condition. In patients with RA, the Disease Activity Score in 28joints (DAS28) was applied, with scores 3.2–5.1 indicating moderate activity and ≥ 5.2 high disease activity [17]. For patients with PsA, diseaseactivity was evaluated using the Disease Activity Index for Psoriatic Arthritis (DAPSA), classified as moderate activity (14–27), and high activity (≥ 28) [9]. In patients with Pso, disease severity was assessed using the Psoriasis Area and Severity Index (PASI), with scores ≥ 10 indicating moderateto high disease activity [7]

FIB-4 in the high-risk category remained independently associated with the presence of advanced fibrosis in almost all the models evaluated, with OR ranging from 5 to 19 and *p* values < 0.01. The only exception was observed in the model adjusted for moderate/high disease activity, in which this association lost statistical significance, possibly due to the small number of events and overlapping effects between variables.

In addition to FIB-4, the variables independently associated with advanced fibrosis after adjustment for confounding factors included BMI, obesity, severe steatosis (grade 3), T2DM, systemic hypertension, waist circumference, and MASLD. Among these, the strongest independent associations were observed for obesity, T2DM, and severe steatosis, all of which are metabolic risk factors. Notably, MASLD and steatosis were not significantly associated with advanced fibrosis in the univariate model but became statistically significant in the multivariable model, suggesting that their effects were initially masked by confounding factors such as other metabolic risks. On the other hand, the variables that were not independently associated with advanced fibrosis after adjustment included sex, disease duration, ethnicity, mild/moderate steatosis, cumulative methotrexate dose, High Density Lipoprotein (HDL), Erythrocyte sedimentation rate (ESR), and moderate/high disease activity.

In subgroup analyses, a high FIB-4 score was associated with a higher prevalence of advanced fibrosis in RA patients (27.8% *versus* 5.1%, *p* = 0.007). A similar, though non-significant, trend was observed in PsA (50.0% *versus* 21.7%, *p* = 0.269). In Pso patients, no individuals were classified as high-risk by FIB-4, preventing evaluation of its association with advanced fibrosis in this subgroup. Due to the small number of events after stratification, these results are considered exploratory.

## Discussion

In this multicenter cross-sectional study, we show that FIB-4 demonstrates potential utility for ruling out advanced hepatic fibrosis in methotrexate users younger than 65 years, while the apparent fibrosis risk is largely driven by confounding factors, particularly metabolic ones. Importantly, we also defined an exploratory, methotrexate-specific FIB-4 cutoff to support better risk stratification in clinical practice.

Chronic methotrexate use has historically been implicated in hepatotoxicity via multiple proposed mechanisms, including oxidative stress, mitochondrial dysfunction, impaired nucleic acid synthesis due to inadequate folic acid supplementation, and ferroptosis, ultimately promoting hepatocyte death and liver fibrosis [1–3, 5, 25–28]. However, recent studies [1, 3, 4, 25, 29], in line with our data, have shown a low incidence of liver injury with chronic low doses or even a lack of correlation between cumulative methotrexate dose and advanced liver fibrosis. In fact, fibrosis progression is influenced by several other factors, including the presence and severity of comorbidities, genetic predisposition, and environmental drivers [16, 26, 30]. Among these, the risk factors most strongly associated with hepatotoxicity in methotrexate users are alcohol consumption, obesity, T2DM, MASLD, and folate deficiency [3, 6]. 

Indeed, pathophysiological evidence shows that patients with MASLD have an increased risk of methotrexate-induced hepatotoxicity [1, 2, 31]. Our findings demonstrate that metabolic disorders act not only as cofactors that potentiate methotrexate-associated liver injury, but also as independent determinants of liver fibrosis, reinforcing their central role in fibrosis risk irrespective of methotrexate exposure. This is particularly relevant given that MASLD is the most common chronic liver disease in the world, affecting approximately 25% of the population [6]. Notably, estimates indicate that the prevalence of advanced liver fibrosis is 6–19% among individuals with T2DM and 4–33% among obese individuals [6, 14]. 

Not only do metabolic comorbidities play an important role in the development of liver fibrosis, but also are associated with disease burden and outcomes in RA, PsA and Pso, since adipose tissue acts as an active inflammatory organ, contributing to systemic inflammation, worsening disease activity and reducing response to therapy. This highlights the importance of integrated management of both inflammatory disease and metabolic health [32, 33]. 

Consistent with the results from this study, Atallah et al. (2023) [16] conducted a multicenter cohort study including 999 patients with RA or Pso and similarly found no association between methotrexate exposure or cumulative dose and liver fibrosis assessed by FibroScan^®^ and Enhanced Liver Fibrosis score (ELF). In contrast, T2DM emerged as the main risk factor for fibrosis, supporting our observation that metabolic factors play a dominant role in hepatic fibrosis risk.

Additional evidence supports the notion that metabolic dysfunction, rather than methotrexate exposure, is the main driver of liver disease in patients with PsA. In a large prospective cohort of 1,314 patients, Kharouf et al. (2025) [34] found that liver fibrosis developed in 11.7% of participants over a median follow-up of 5.6 years, with higher BMI and T2DM emerging as independent predictors, while cumulative methotrexate dose showed no significant association. Consistent with these findings, Meridor et al. (2026) [35] reported a high prevalence of MASLD-related clinically significant liver disease (61.8%) in a cross-sectional study of 89 patients with PsA, identifying obesity and hypertension as key associated factors and again finding no relationship with methotrexate exposure. Based on the high negative predictive value of FIB-4, the authors proposed a screening strategy incorporating FIB-4 followed by transient elastography to improve early detection, risk stratification, and clinical management of liver disease in this population. In light of these findings, the primary focus should perhaps be on reducing metabolic risks in patients receiving methotrexate—particularly T2DM and obesity — which are clearly associated with liver fibrosis. Accordingly, updating monitoring guidelines for patients on methotrexate to take comorbidities into account may be beneficial.

Population-based studies [5] indicate a higher prevalence of liver-related outcomes in patients with Pso and PsA compared to RA, demonstrating that the underlying inflammatory disease itself may be an independent risk factor for liver fibrosis, possibly by lowering the threshold for methotrexate hepatotoxicity. No correlation between disease activity and advanced fibrosis was found in this study, possibly because this analysis may have been limited by the small number of individuals with moderate to severe activity, as most of the sample was in disease remission.

Although FIB-4 is recognized as the most validated and best-performing noninvasive score for excluding advanced liver fibrosis and is widely accessible due to its reliance on simple variables [1, 6, 11, 12], its diagnostic performance is strongly influenced by age. The score was originally developed in a population with a mean age of approximately 40 years [19], and its specificity declines markedly in older individuals, dropping to below 30% among those over 65 years of age [36]. This age-related limitation was confirmed in the present study, in which FIB-4 demonstrated poor discriminatory performance on ROC curve analysis in patients aged 65 years or older. Taken together, these data suggest that FIB-4 has limited accuracy for evaluating advanced liver fibrosis in this age group, underscoring the need for complementary or alternative fibrosis assessment strategies to ensure reliable risk stratification.

In addition to age, the accuracy of FIB-4 is highly dependent on its cutoff values, which vary according to the underlying liver disease and clinical context, given that the original thresholds were derived from patients with HIV–hepatitis C coinfection and may be further affected by extrahepatic conditions influencing transaminase levels or platelet counts [6, 19]. A cutoff of 1.3 (for individuals < 65 years old) or 2.0 (for individuals ≥ 65 years old) effectively rule out advanced fibrosis in MASLD and HIV-hepatitis C coinfection, whereas higher thresholds are associated with progressively increased liver stiffness [37, 38]. Importantly, this study is, to our knowledge, the first to establish a FIB-4 cutoff to stratify the risk of advanced fibrosis in patients with RA, PsA or Pso receiving methotrexate therapy. This study-derived cutoff of 1.62, previously unreported in this clinical context, demonstrated favorable overall performance, including reasonable accuracy and a high negative predictive value in patients younger than 65 years. Although the high negative predictive value was influenced by the relatively low prevalence of advanced fibrosis in the study population, the findings suggest that this threshold may be a useful tool for excluding advanced fibrosis and supporting risk stratification in clinical practice. Future external validation is warranted to confirm its performance across diverse populations.

A two-step strategy using FIB-4 followed by elastography could substantially reduce unnecessary elastography exams and specialist referrals, although this approach may miss a small proportion of patients with increased liver stiffness, supporting the use of FIB-4 primarily as a risk stratification tool [6]. 

This study has several important strengths. First, it was conducted in a population of patients followed within the Brazilian public health system, a setting in which access to elastography is limited, and liver biopsy is not commonly feasible. Demonstrating the reliability of FIB-4 in this context represents a meaningful achievement, as it supports the use of a low-cost, widely available tool to guide clinical decision-making where advanced diagnostic resources are scarce. In addition, the multicenter design enhances the generalizability of the findings, while the identification of a methotrexate-specific FIB-4 cutoff and the consideration of metabolic confounders provide clinically relevant insights that may improve risk stratification and optimize referrals to specialized Hepatology care.

Although this study has notable strengths, certain limitations must be considered, including a relatively restricted sample size due to logistical challenges in accessing the site where the FibroScan^®^ was located; nevertheless, the sample was sufficient to identify significant associations. Due to the small number of cases of advanced fibrosis, subgroup analyses were considered exploratory and residual confounding among correlated metabolic factors may have occurred. Potential operator dependence and technical limitations inherent to elastography were recognized but mitigated using experienced examiners, with only a single examination excluded due to severe obesity. In addition, advanced fibrosis was defined using elastography rather than histology, which, although appropriate in a real-world setting, should be acknowledged as a limitation due to the lack of histopathological confirmation.

## Conclusions

Our results support FIB-4 as an accessible and potentially useful tool for estimating the risk of advanced liver fibrosis, particularly where access to elastography remains limited. FIB-4 proved especially useful for excluding advanced fibrosis in individuals younger than 65 years, and notably, this study identifies an exploratory population-specific cutoff of 1.62 - a threshold not previously established in the literature for methotrexate-treated patients with RA, PsA, and PsO. This novel cutoff demonstrated reasonable accuracy as an initial screening strategy, enhancing risk stratification and supporting more targeted and appropriate referral to specialized hepatology care. However, its low positive predictive value underscores the need for a stepwise diagnostic approach incorporating elastography when indicated. Importantly, advanced liver fibrosis, identified in 11% of the sample, was not associated with the underlying rheumatologic or dermatologic disease, inflammatory activity, or cumulative methotrexate dose, suggesting that methotrexate-related hepatic risk may have been overestimated in earlier studies. Instead, metabolic factors, particularly obesity, T2DM, and severe hepatic steatosis, emerged as the main independent factors associated with advanced fibrosis, highlighting the importance of prioritizing metabolic risk assessment and management to improve patient care and optimize healthcare resource utilization.

## Data Availability

The datasets used and/or analysed during the current study are included within the article.

## Abbreviations

ALT: Alanine Aminotransferase
AST: Aspartate Aminotransferase
AUC: Area Under the Curve
BMI: Body Mass Index
CAP: Controlled Attenuation Parameter
CI: Confidence Interval
DAS28: Disease Activity Score in 28 Joints
DAPSA: Disease Activity Index for Psoriatic Arthritis
ELF: Enhanced Liver Fibrosis (score)
ESR: Erythrocyte Sedimentation Rate
F0–F4: Fibrosis stages 0 to 4
F3/F4: Advanced liver fibrosis (fibrosis stages 3 or 4)
FIB-4: Fibrosis-4 Index
HDL: High-Density Lipoprotein
HIV: Human Immunodeficiency Virus
IQR: Interquartile Range
kPa: Kilopascals
MASLD: Metabolic Dysfunction–Associated Steatotic Liver Disease
MTX: Methotrexate
NPV: Negative Predictive Value
OR: Odds Ratio
PASI: Psoriasis Area and Severity Index
PPV: Positive Predictive Value
PsA: Psoriatic Arthritis
Pso: Psoriasis
RA: Rheumatoid Arthritis
REC: Research Ethics Committee
ROC: Receiver Operating Characteristic
SD: Standard Deviation
S0–S3: Steatosis grades 0 to 3
T2DM: Type 2 Diabetes Mellitus
TNF: Tumor Necrosis Factor
VCTE: Vibration-Controlled Transient Elastography
WC: Waist Circumference
